# Exploring the Role of Immune System and Inflammatory Cytokines in SARS-CoV-2 Induced Lung Disease: A Narrative Review

**DOI:** 10.3390/biology12020177

**Published:** 2023-01-22

**Authors:** Claudio Tirelli, Mara De Amici, Cristina Albrici, Sabrina Mira, Giulia Nalesso, Beatrice Re, Angelo Guido Corsico, Michele Mondoni, Stefano Centanni

**Affiliations:** 1 Respiratory Unit, ASST Santi Paolo e Carlo, Department of Health Sciences, University of Milan, 20142 Milan, Italy; 2 Immuno-Allergology Laboratory of Clinical Chemistry and Department of Pediatrics, IRCCS Policlinico San Matteo University Hospital, 27100 Pavia, Italy; 3 Pulmonology Unit, Department of Medical Sciences and Infectious Diseases, IRCCS Policlinico San Matteo University Hospital, 27100 Pavia, Italy

**Keywords:** SARS-CoV-2, COVID-19, cytokine storm, immune system, immunotherapy, vaccine, ARDS, innate immune system, adaptive immune system

## Abstract

**Simple Summary:**

Severe acute respiratory syndrome coronavirus 2 (SARS-CoV-2) is responsible for the pandemic coronavirus disease-2019 (COVID-19), which can lead to severe pneumonia and acute respiratory distress syndrome (ARDS). The immune system plays a key role in the defense against COVID-19. Nonetheless, if dysregulated and hyperactivated, the immune response can trigger the deleterious “cytokine storm”. Multiple studies are aimed at finding potential immunomodulators to rebalance the immune response against the virus, thus limiting its dangerous effects. Moreover, the development of vaccines against SARS-CoV-2 has represented a global challenge. The aim of this narrative review is to describe the state-of-the-art knowledge on the immune system response in COVID-19 and to underline the main therapeutic strategies and the key role of vaccines.

**Abstract:**

Severe acute respiratory syndrome coronavirus 2 (SARS-CoV-2) is the causative pathogen of coronavirus disease 19 (COVID-19). COVID-19 can manifest with a heterogenous spectrum of disease severity, from mild upper airways infection to severe interstitial pneumonia and devastating acute respiratory distress syndrome (ARDS). SARS-CoV-2 infection may induce an over activation of the immune system and the release of high concentrations of pro-inflammatory cytokines, leading to a “cytokine storm”, a recognized pathogenetic mechanism in the genesis of SARS-CoV-2-induced lung disease. This overproduction of inflammatory cytokines has been recognized as a poor prognostic factor, since it can lead to disease progression, organ failure, ARDS and death. Moreover, the immune system shows dysregulated activity, particularly through activated macrophages and T-helper cells and in the co-occurrent exhaustion of lymphocytes. We carried out a non-systematic literature review aimed at providing an overview of the current knowledge on the pathologic mechanisms played by the immune system and the inflammation in the genesis of SARS-CoV-2-induced lung disease. An overview on potential treatments for this harmful condition and for contrasting the “cytokine storm” has also been presented. Finally, a look at the experimented experimental vaccines against SARS-CoV-2 has been included.

## 1. Introduction

Coronaviruses are enveloped RNA viruses of the genus of the *Coronaviridae* family [1]. Coronaviruses have been identified in both human and non-human species. While in human species, before the emergence of severe acute respiratory syndrome coronavirus (SARS-CoV), these viruses have always been considered the cause of mild–moderate infections of the respiratory system (the common winter–autumnal “colds”) and, rarely, of pneumonia, in non-human species, coronaviruses are often responsible for severe epizootics infections of the enteric and respiratory tract.

In 2002, a novel coronavirus originated from a mutation in a non-human host (bats) gaining the ability to affect humans. This virus, denominated SARS-CoV, was identified as the pathogenic agent of the SARS outbreak that occurred in Guangdong Province of China [2]. SARS was the most severe human disease caused by a coronavirus. The transmission of SARS-CoV was relatively inefficient; indeed, its spread occurred only through direct contact with infected individuals, with negligible infectivity during an incubation state. The outbreak was largely contained within households and healthcare settings. 

A novel human coronavirus, named Middle East Respiratory Syndrome-CoV (MERS-CoV), emerged in the Middle East in 2012. The MERS-CoV clinical spectrum ranged from asymptomatic infection to acute respiratory distress syndrome, septic shock, multi-organ failure and death, with a mortality rate of 35% [1]. The outbreak was controlled by the end of 2014.

In December 2019, another novel coronavirus, called SARS-CoV-2, capable of transmitting to humans and highly pathogenic, emerged in the Wuhan region of China. The virus is responsible for severe pneumonia and acute respiratory distress syndrome (ARDS). SARS-CoV-2 disease, also called coronavirus disease-2019 (COVID-19), was declared a pandemic by the World Health Organization (WHO) on 11 March 2020 [2].

SARS-CoV-2 can transmit with a more efficient pattern than SARS-CoV and MERS-CoV, also during the asymptomatic period of incubation [3]. 

SARS-CoV-2 infection can manifest variably from asymptomatic forms to severe pneumonias with ARDS, needing hospitalization in medical wards and intensive care units (ICUs) [4]. 

The disease commonly manifests with non-specific symptoms, including fatigue, cough and fever. Dyspnoea with hypoxemic respiratory failure is the leading cause of hospital admission. Chest radiography and computed tomography usually show bilateral interstitial pneumonia, with lung consolidations, ground glass opacifications and, sometimes, areas of organizing pneumonia. COVID-19 pneumonia can drastically progress towards ARDS. Moreover, the disease can also manifest with non-respiratory involvement, ranging from anosmia, ageusia, to severe neuroinflammatory neurovascular disease (i.e., meningitis and encephalitis), and to preeclampsia in pregnant women. Respiratory failure in COVID-19 may be related not only to lung parenchyma damage, but also to a state of hyper-coagulation, which might lead to microangiopathy and pulmonary embolism (PE), with a reported overall cumulative incidence of 24%. Laboratory testing classically shows non-specific findings, i.e., lymphocytopenia, and elevated levels of lactate dehydrogenase and C-reactive protein [5]. 

Several studies have demonstrated that the immune system plays a key role in COVID-19. In particular, the immune response, both innate and adaptive, contributes not only to the defense against SARS-CoV-2, but also—if dysregulated and hyperactivated—can lead to deleterious effects and the “cytokine storm”, which is the basis of the more severe clinical forms of COVID-19. Cytokine storm syndrome has been described in several immune-based pathologies and its role in COVID-19 has been thoroughly investigated. It is characterized by elevated serum concentrations of pro-inflammatory cytokines (i.e., IL-6; Tumor necrosis factor Alpha-TNF-α) and is associated to fatal multi-organ failure [6]. 

The aim of this narrative review is to describe the state-of-the-art knowledge on the immune system response in COVID-19 and to underline the main therapeutic strategies and the key role of vaccines.

## 2. Methods

We carried out a non-systematic, narrative literature review. The most relevant articles were retrieved through the search engine PubMed. Suitable original articles and reviews written in English were selected. Only studies on adult human beings were considered. The following keywords were combined to create ad hoc strings: COVID-19; SARS-CoV-2; cytokines; cytokine storm; innate immune system; adaptive immune system; T cell; B cell; immunotherapy; antibodies; vaccines; lymphopenia; inflammasome.

## 3. Results

### 3.1. SARS-CoV-2 Infection

Coronaviruses (CoV) are single-stranded RNA viruses that infect various vertebrates. SARS-CoV-2 is a new beta-coronavirus that emerged at the end of 2019 in the Hubei province of China. It is the cause of COVID-19 [7]. Human CoVs are transmitted primarily through respiratory droplets, but aerosol, direct contact with contaminated surfaces and fecal–oral transmission were also reported during the SARS epidemic [8] SARS-CoV-2 is mainly transmitted in humans through respiratory droplets, since the presence of replicating SARS-CoV-2 has been demonstrated in both the upper and lower respiratory tract [9]. Once SARS-CoV-2 enters the host, it infects target cells to replicate and spread novel viral particles. 

SARS-CoV-2 is an enveloped, positive-sense single-stranded RNA virus. Its genome comprises 14 open reading frames (ORFs), two-thirds of which encode 16 nonstructural proteins (nsp 1–16) that compose the replicase complex. The remaining one-third of the genome includes nine accessory ORFs and the genes encoding for four fundamental structural proteins: spike (S), envelope (E), membrane (M) and nucleocapsid (N). SARS-CoV-2 enters host cells through the interaction of the S protein with the human angiotensin converting enzyme 2 (ACE2) [10]. The cellular entry of SARS-CoV-2 requires direct contact of the receptor-binding domain (RBD) of the S protein with ACE2 and S protein priming by cleavage at the S1/S2 site, so that the S2′ subunit can allow the fusion of viral and cellular membranes. S1/S2 polybasic cleavage site is proteolytically cleaved by cellular cathepsin L and the transmembrane protease serine 2 (TMPRSS2). TMPRSS2 facilitates viral entry at the plasma membrane surface, whereas cathepsin L activates SARS-CoV-2 Spike in endosomes and can compensate for entry into cells that lack TMPRSS2 [11]. Once the genome is released into the host cytosol, ORF1a and ORF1b are translated into viral replicase polyproteins (pp1a and pp1b), which are cleaved into individual nonstructural proteins via host and viral proteases; these form the RNA-dependent RNA polymerase. Replication begins in virus-induced double membrane vesicles derived from the endoplasmic reticulum. Here, the incoming positive-strand genome translation results in both structural proteins and accessory proteins that are inserted into the endoplasmic reticulum–golgi intermediate compartment for virion assembly. Finally, subsequent positive-sense RNA genomes are incorporated into newly synthesized virions, which are secreted from the plasma membrane [12]. Due to their expression of ACE2 and TMPRSS2, alveolar epithelial cells, vascular endothelial cells and alveolar macrophages are among the preferred targets for SARS-CoV-2 infection and subsequent replication [13].

### 3.2. Innate Immune Response to SARS-CoV-2 Infection and COVID-19

Innate immunity represents the first line of defense against microorganisms. It is composed of numerous cellular elements, including macrophages, natural killer cells, monocytes, dendritic cells and neutrophils. The complement system, together with the coagulation–fibrinolysis system and interferons, also contribute to the innate immune response. Among their protective functions, these elements can limit the proliferation of infective viruses such as SARS-CoV-2 [14]. 

*Interferon*: Interferons (IFN) constitute the major first line defense against viruses. Particularly, IFN types I and III play an important defensive role. They are produced by the interaction between specific viral elements and pattern recognition receptors (PRR), which are expressed on the membranes of the innate immune system cells. This interaction activates the nuclear factor kappa-light-chain-enhancer of activated B cells (NF-kB) and the Janus kinase–signal transducer and activator of transcription (JAK-STAT) pathways, with the consequent production of IFN type I, IFN type III and proinflammatory cytokines, such as interleukin (IL)-6 and tumor necrosis factor (TNF)-α, which can induce viral resistance, interfering with viral replication. The receptors of IFN type I are present in all somatic cells, while IFN type III receptors can be retrieved in epithelial cells, neutrophils, dendritic cells, macrophages and B lymphocytes [14]. Curiously, it has been described that during SARS-CoV-2 infection, a dysregulation of the immune response mediated by IFN can occur. Some studies have shown that patients with severe COVID-19 disease had a reduced or absent IFN type I activity (characterized by the absent production and activity of IFN-β and low activity of IFN-α), compared to patients with mild and moderate disease. Several hypotheses have been advanced to explain this variability of response to SARS-CoV-2 infection, including comorbidities and genetic susceptibility; however, the precise mechanisms have not been fully clarified [15]. One study conducted in 987 patients with life-threatening COVID-19 pneumonia showed that 101 patients had neutralizing immunoglobulin G (IgG) against IFN type I. These autoantibodies neutralized the activity of IFN type I, causing serious illness secondary to the inactivation of the innate immune response [16]. On their surface Toll-like receptors (TLR), innate immune system cells express a variety of PRRs that are able to detect pathogen-associated molecular patterns (PAMPs), which are located in both the intracellular and extracellular environment. These interactions stimulate the expression of transcription factors such as NF-kB, which induces the production of pro-inflammatory cytokines. Moreover, within the cytosol of these cells there is another family of Nucleotide-binding domain Leucine-rich Repeat proteins (NLR) that specifically recognizes endogenous Damage-Associated Molecular Patterns (DAMPs), which are endogenous molecules that are released from damaged or dying cells [17]. The bond between DAMPs and NLRs causes their activation and triggers the formation of inflammasome [18]. 

*Inflammasome*: The inflammasome is a cytosolic multimolecular complex that promotes the innate immune system to recognize infective agents and molecules from host proteins. The inflammasome plays a role not only in infectious disease, but also in cancers and inflammatory diseases [19]. Among all the inflammasomes, the nucleotide-binding oligomerization domain-like receptor containing pyrin domain 3 (NLRP3) inflammasome is the best studied and one of the most involved in COVID-19. NLRP3 is composed of NLRP3 protein, a leucine-rich repeat (LRR) domain with a central nucleotide-binding domain (NACHT), and a pyrin domain ((PYD), an adapter apoptosis-associated speck-like protein containing a caspase recruitment domain (ASC) and pro-caspase-1 [20]. In COVID-19, the acute immune response is mainly driven by monocytes and alveolar macrophages. They are activated by PAMPs and DAMPs, which are released by type 2 pneumocytes infected by the virus [21]. Indeed, SARS-CoV-2 has a particular tropism for ACE2 receptor on type 2 pneumocytes, which participate in the innate response by expressing TLRs [22]. PAMPs and DAMPs are recognized by TLR and activate some transcription factors, such as NF-kB, which upregulates the expression of NLRP3 inflammasome components, proinflammatory cytokines and pro-caspase-1. Later, this process leads to the activation of NLRP3 by oligomerization and the subsequent assembly of a complex composed of NLRP3, ASC and pro-caspase-1, which causes the activation of pro-caspase-1 in caspase-1. Caspase-1 induces the production of pro-inflammatory cytokines, converting pro-IL-1β and pro-IL-18 into mature IL-1β and IL-18, triggering the inflammatory cytokine storm and ultimately inducing cell death and the rapid elimination of microbial elements by pyroptosis [17,20]. Normally, all these processes play a protective role in contributing to the elimination of the virus in the early stages of infection, thus, avoiding the development of severe forms [17,18]. SARS-CoV-2 replication, however, can alter the integrity of the cell membrane and the ion fluxes through it, enhancing inflammation and cell death. More in detail, the entry of SARS-CoV-2 into the cells may compromise the normal physiological homeostasis as a response to the dysfunctional K^+^ efflux and Ca^2+^ influx induced by viroporins, which stimulate the activation of NLRP3 and a hyperproduction of proinflammatory cytokines [20]. Particularly, the release of IL-1β and IL-18 stimulates the activation of innate immune cells and the overproduction of other inflammatory cytokines, such as IL-6, TNFα, IL-8 and IL-10, paving the way to the harmful cytokine release syndrome, the basis for the development of acute respiratory distress syndrome (ARDS), multiorgan failure and coagulopathy [21,23]. 

*The complement system* is a crucial trigger for the pro-inflammatory response in COVID-19. Worth mentioning is the mannose-binding lectin (MBL), which plays a pivotal role in innate immunity, interacting with the surface sugars of a wide series of microorganisms—including SARS-CoV-2—as a PRR. MBL seems to be deputed to three principal roles: it activates the lectin complement pathway, then it promotes opsonin phagocytosis and, at last, it modulates inflammation [24]. Genetic polymorphisms of MBL, resulting in lower levels or a reduced expression of functional MBL, might predispose to bacterial and viral infections, and in the past they have been correlated to a major susceptibility to SARS-CoV infection [25]. MBL seems able to bind SARS-CoV through the carbohydrate recognition domains to stimulate C4 deposition in the virus, and, in experimental models, seems to reduce its capacity for infection [26]. The presence of mannose-rich glycans in the S1 region of SARS-CoV-2 has raised the hypothesis that glycan recognition and binding to MBL may inhibit S1-ACE interaction, thus limiting SARS-CoV-2 infection [24]. 

*Neutrophils and NETosis:* Neutrophils are the largest population of myeloid leukocytes. During infections, neutrophils immediately migrate from the bloodstream to the site of infection, providing a “first line” defense against pathogens [27]. Neutrophils are rich in granules containing antimicrobial agents capable of killing pathogens through phagocytosis. Bactericidal enzymes can also be released from the cells during degranulation. Finally, through a process named NETosis, neutrophils can release neutrophil extracellular traps (NETs), which consist of extracellular webs of neutrophil-modified chromatin, histones and microbiocidal proteins released from granules and cytoplasm in response to the activation of neutrophils by PPRs or chemokines, in order to contrast the infections [14,27]. Currently, two fundamentally different forms of NETosis have been described: vital NETosis, which maintains the cell viability and many of its effector functions, and suicidal NETosis. This is a special form of programmed cell death characterized by the release of granule components into the cytosol, as well as chromatin de-condensation, which is associated with histone modification. During NETosis, like in apoptosis, coordinated changes in the nucleus and in the cytoplasm occur without requiring the activation of caspases. In addition, NETosis is accompanied by a disturbance of the insulating properties of the plasma membrane. The signaling mechanisms of NETosis may include activation of phosphoinositide-3-kinase (PI3K), which also controls the autophagy induction, not requiring the assembly of autophagosomes. In the respiratory tract, NETosis contributes to the protection against infection by increasing the viscosity of the mucus and favoring the killing of pathogens [27]. Veras et al. found that circulating neutrophils infected with SARS-CoV-2 released high levels of NETs, and that an increased concentration of NETs can be retrieved in plasma, tracheal aspirate and lung tissue specimens from COVID-19 patients [28]. In COVID-19, it has been hypothesized that NETosis can be stimulated by damaged epithelial and endothelial cells, activated platelets and inflammatory cytokines [27]. This is sustained by the detection of an increased level of NETosis markers (cell-free DNA, mieloperoxidase-DNA complexes and citrullinated histone H3) and markers of cell death (i.e., lactate dehydrogenase) in COVID-19 patients. While NETs have microbicidal activity, their sustained release can also trigger inflammation, playing a central role in cytokine storm, and facilitate micro-thrombosis, thus, resulting in multi-organ damage (pulmonary, cardiovascular and renal) [29]. 

### 3.3. Adaptive Immune Response to SARS-CoV-2 Infection and COVID-19

**Figure 1:**
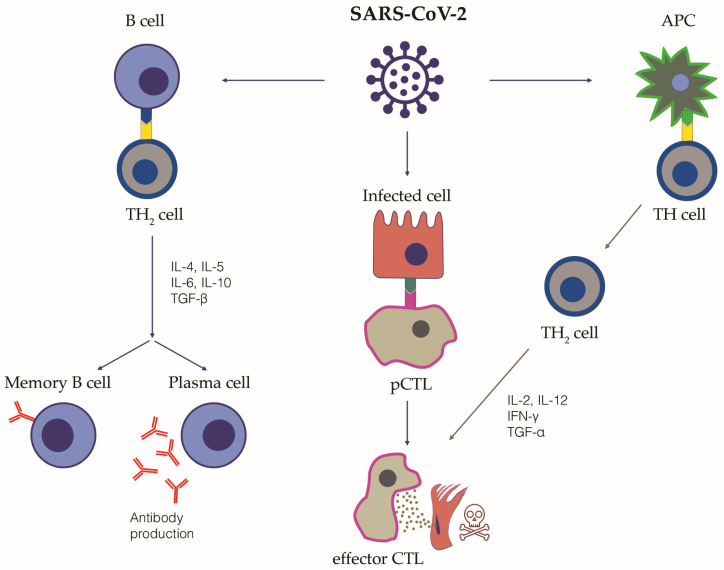
The adaptive immune response to SARS-CoV-2 infection. This figure schematically summarizes the adaptive immune response following SARS-CoV-2 infection. The cellular pathways and the relative key cytokines are highlighted. Following their activation by interaction with the antigen-presenting cells (APC), TH_2_ lymphocytes are able to release cytokines and activate B cells and cytotoxic T lymphocytes (CTL), which exert their cytotoxicity on infected cells. Promoting CTL can also be directly activated by the interaction with SARS-CoV-2 infected cells. Once activated, B cells can differentiate into plasma cells, releasing specific anti SARS-CoV-2 neutralizing antibodies, and Memory B cells. The immunological memory towards SARS-CoV-2 is mediated both by virus-specific memory B lymphocytes, but also virus-specific memory CD4+ and CD8+ T lymphocytes, Natural Killer T lymphocytes and circulating antibodies. TH, T Helper cell; APC, antigen-presenting cell, CTL, cytotoxic T lymphocyte; IL, interleukin; TGF, transforming growth factor; IFN, interferon.

The adaptive immune response is generated by T and B lymphocytes. T lymphocytes are responsible for the cell-mediated immune response and are distinguished in cytotoxic T lymphocytes, T helper lymphocytes, suppressor T lymphocytes and memory T lymphocytes. B lymphocytes can differentiate into specific antibody-producing plasma cells, namely short-lived and long-lived plasma cells (Figure 1).

*B lymphocytes and antibodies production*: B cells are stimulated by circulating IL-6 and TNF-α [30]. Notably, in COVID-19, a decrease in memory B cells and an increase in hyperactivated plasmablasts has been observed, with a consequent oligoclonal production of antibodies [31,32]. Highly-specific circulating SARS-CoV-2 neutralizing antibodies directed against the spike protein provide protective immunity. Nonetheless, a significant amount of the circulating antibodies are not neutralizing, but probably directed against SARS-CoV-2 antigens other than the spike protein. After SARS-CoV-2 infection, IgM antibodies can be detected between days 8 and 12, and their levels drop and tend to disappear by week 12. On the contrary, specific IgG antibodies typically start to be detected from day 14 and last for a longer time. Their titer has been correlated with the severity of the infection and the viral load [33]. In patients who suffered from mild disease or asymptomatic infection, the humoral response more frequently tends to decrease earlier [34]. Intriguingly, it has been hypothesized that antibodies might not play a crucial role in the immune response to SARS-CoV-2, as it has been described in some case series of patients with humoral immune deficiency but preserved innate immune response, showing good recovery from the infection [35,36]. This supports the key role of a rapid and effective innate immune response in SARS-CoV-2 infection. Moreover, higher levels of specific IgA, IgM and IgG antibodies have been found in patients with worse clinical outcomes, possibly due to the harmful effects of the genesis of pathogenic immune complexes [37,38]. The activation of complement secondary to the presence of circulating IgM and IgG immune complexes may lead to intravascular coagulation, whereas in local tissues immune complexes secondary to the presence of IgA antibodies might cause inflammation and micro-thrombosis [37,38]. In the alveoli, the immune complexes binding to the Fc receptors of the macrophages induce the release of pro-inflammatory cytokines, thus enhancing inflammation and tissue damage [39]. Intriguingly, epitopes of SARS-CoV-2 similar to self-constituents can favor immunological cross-reactivity and, thus, the development of an autoimmune response [40]. Finally, immunological memory plays a key role in the development of long-lasting protective immunity [41]. Immunological memory is mediated by virus-specific memory B lymphocytes, but also virus-specific memory CD4+ and CD8+ T lymphocytes, Natural Killer T lymphocytes (NKT) and circulating antibodies. Immunological memory towards SARS-CoV-2 can be activated by the direct infection (natural immunity), vaccination or hybrid immunity, which is the combination of infection and vaccine-induced immunity. 

*T lymphocytes:* T lymphocyte subsets contributing to protective immunity to SARS-CoV-2 infection include: (1) CD8+ T cells, particularly virus-specific CD8+ Cytotoxic T Lymphocyte; (2) several subsets of CD4+ T helper cells—namely Th1, Th2, Th17 and T Follicular Helper—each associated with a distinctive pattern of cytokine secretion, transcription factors and differentiation profiles [41]. SARS-CoV-2 specific CD4+ T lymphocytes also include a subset of cytotoxic cells that can exert direct antiviral function. Follicular Helper T cells are mainly resident in the germinal center of lymphoid organs where rapidly proliferating B cells undergo somatic mutation that allow the secretion of highly specific memory antibodies. Follicular Helper T cells play a role of paramount importance in orchestrating the development and maturation of the antibody response, promoting the B cell differentiation into memory B cells and plasma cells secreting high-affinity and high-specificity antibodies [42]. Increased activation of Th17 together with a decreased activity of CD4+ regulatory T lymphocytes (Treg) has been observed in severe COVID-19 patients, while the imbalanced ratio Th17/Treg is strongly associated with hyperinflammation, lung damage and disease pathogenesis. CD4+ T lymphocytes are able to release cytokines, and activate B cells and CD8+ T lymphocytes, which exert their cytotoxicity on infected cells [43]. Cytotoxic T lymphocytes are particularly active during the acute phase of COVID-19, though it is not rare to observe a reduction in CD8+ T cells in severe cases of COVID-19 with a consequent neutrophilia [44]. Peripheral lymphopenia has been thoroughly described in COVID-19, and it has been hypothesized to be an immune-suppressive phase consequent to the initial hyperinflammation phase, although the exact mechanisms beyond lymphopenia are not yet fully understood [45]. A direct cytopathic effect of SARS-CoV-2 on lymphocytes might be excluded in the absence of ACE-2 receptors on these cells, while enhanced apoptosis driven by TNF-α, or SARS-CoV-2 alternative mechanisms of lymphocytes invasion driven by CD147, seem more plausible [46,47]. Notably, if persistent, lymphopenia may facilitate bacterial co-infection in COVID-19, with poor prognostic outcomes [10]. During the convalescent phase, T lymphocytes mainly show a memory phenotype. Intriguingly, several studies have described a potential immunopathological role for Th17 lymphocytes in COVID-19 [48]. These cells release IL-17, a key cytokine which can contribute to the cytokine storm, mediating the activation of the innate immune system, the release of pro-inflammatory cytokines (IL-1, IL-6, TNF-α) and the stimulation of neutrophils, macrophages, monocytes and dendritic cells. It is also worth mentioning the important role of local tissue immunity, mediated by both natural and adaptive immunity, in protecting from COVID-19. For instance, tissue resident memory T cells, both CD4+ and CD8+, are present at measurable frequency in the lungs, and SARS-CoV-2-specific CD8+ T lymphocytes have been identified in the lungs and in thoracic lymph nodes, sometimes at a higher frequency than in the peripheral blood. Of interest, CXCR6+ CD8+ T lymphocytes in the lungs have been associated with long-lasting COVID-19 inflammation and might play a role in long COVID-19 [49]. 

### 3.4. Cytokine Storm

**Figure 2:**
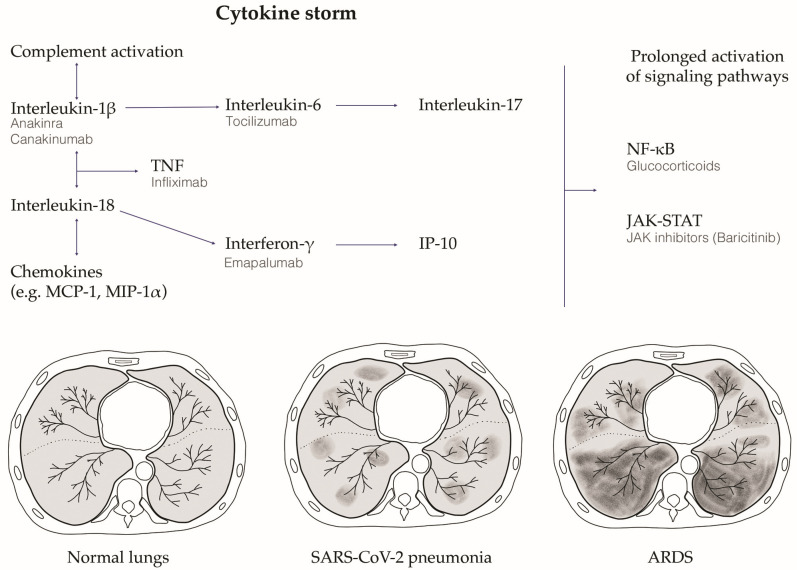
The SARS-CoV-2 infection induced cytokine storm and the relative lung damage. After SARS-CoV-2 infection, an innate and adaptive immune response is activated, which leads to the release of high amounts of pro-inflammatory cytokines (the cytokine storm schematically reported in the figure). This process clinically manifests first with respiratory failure and interstitial pneumonia, which are typically characterized by diffuse ground glass opacifications detected using high-resolution computed tomography. The more this process auto-amplifies, the higher is the risk for the patient to develop acute respiratory distress syndrome (ARDS) and multiorgan failure, which can finally lead to death. The figure schematically depicts axial slices from high-resolution computed tomography (HRCT) showing normal lungs, SARS-CoV-2 interstitial pneumonia with the typical ground glass opacifications, and the end-stage ARDS. Therapeutic strategies experimented in COVID-19 targeting the cytokine storm pathways are also depicted. TNF, tumor necrosis factor; NF-kB, nuclear factor kappa B; JAK-STAT, Janus kinase—signal transducers and activators of transcription; Monocyte Chemoattractant Protein-1, MCP-1; Macrophage Inflammatory Protein-1 Alpha, MIP-1 alpha; Interferon gamma-induced protein 10, IP-10.

An M1 macrophage and Th1 lymphocyte polarized inflammatory cytokine storm has been proposed as one of the harmful pathobiological mechanisms at the basis of the clinical deterioration in severe COVID-19 [45]. When SARS-CoV-2 replicates in target type 2 pneumocytes, it induces pyroptosis due to its high cytopathic effect [50]. Pyroptosis is an inflammatory form of programmed cell death secondary to viral infections [51], which has been proposed as one of the possible primum movens for the activation of the hyper-inflammation that initiates the cytokine storm cascade [52]. Indeed, pyroptotic lung epithelial cells release both proinflammatory cytokines, such as IL-1β, and the pathogen-associated molecular patterns, such as viral RNA particles, which are detected by the PRRs and IL-1R of pneumocytes and lung macrophages. This process induces the release of high levels of proinflammatory cytokines (i.e., IP10, CCL2, CCL3, CCL4, IFN- γ, IL-6), which attract T cells and macrophages. These cells amplify the inflammatory cascade, induce circular pro-inflammatory feedback, releasing other pro-inflammatory cytokines, and contribute to lung tissue damage. This clinically manifests first with respiratory failure and interstitial pneumonia, which are typically characterized by diffuse ground glass opacifications detected using high-resolution computed tomography. The more this process auto-amplifies, the higher is the risk for the patient to develop ARDS and multiorgan failure, which finally leads to death [53] (Figure 2). 

Recent studies have focused on the potential role that type 2 pneumocytes might play in the induction and maintenance of the dangerous cytokine storm [54]. Intriguingly, it has been observed that type 2 pneumocytes, primarily deputed to regenerate damaged lung tissue and release the surfactant, might also behave as innate immune cells. Indeed, NF-kB may be activated by TLR expressed by type 2 pneumocytes after viral infection [55]. Moreover, infected type 2 pneumocytes can release high levels of IL-1, IL-6, TNF- α, favoring cytokine storm and the chemo-attraction of macrophages, monocytes and T lymphocytes in the damaged lungs. Other studies have demonstrated that hyperinflammatory damage is elicited by a contextual weaker IFN response, particularly of IFN type I and III, although the precise mechanism beyond it is not fully clear [56]. 

Several clinical studies on mortality in COVID-19 have outlined that non-survivor patients typically had elevated serum levels of pro-inflammatory cytokines (IL-2, IL-7, IL-10, G-CSF, IP-10/CXCL10, MCP-1/CCL2, MIP-1a/CCL3 and TNF- α), together with ferritin, C reactive protein and lactate dehydrogenase, which peaked before the development of ARDS [57,58]. 

### 3.5. Therapy: Targeting the Modulation of Cytokine Storm

Since the beginning of the COVID-19 pandemic, scientific research has tried to find effective therapies. Quickly, it became clear that dysregulation of the immune response against SARS-CoV-2 is a major feature of COVID-19, especially in the severest forms. Therefore, multiple studies aimed at finding potential immunomodulators to rebalance the immune response against the virus, thus limiting its dangerous effects. Collaterally, antiviral drugs have been studied with the intention of achieving early clearance of the virus, in order to restrain the cascade of immune dysregulation. This paragraph presents the principal therapeutic agents that have been tested so far (Table 1, Figure 2).

*Corticosteroids* are among the most adopted anti-inflammatory and immune-modulator drugs. They have a widespread inhibitory effect on the immune system and exert anti-inflammatory activity by inhibiting the expression of proinflammatory transcription factors. Particularly, glucocorticoids induce gene transcription and the protein synthesis of nuclear factor kappa B (NF-kB) and lipocortin-1 inhibitors, through which they inhibit the synthesis of downstream proteins in the inflammatory cascade, such as IL-1, IL-6, granulocyte-macrophage colony-stimulating factor (GM-CSF) and inducible cyclooxygenase-2 (iCOX2). By inhibiting the synthesis of Th1 and macrophage pro-inflammatory cytokines, such as IL-1β, IL-2, IL-6, TNF-α and IL-17, glucocorticoids reduce the proliferation, activation, differentiation and survival of T cells and macrophages [59]. Numerous trials have been performed to study the clinical effects of steroids in COVID-19. RECOVERY was the first randomized clinical trial to report that dexamethasone at a dose of 6 mg daily for 10 days reduces the 28-day mortality rate in patients hospitalized for COVID-19 requiring oxygen therapy and/or invasive mechanical ventilation [60]. The effect of methylprednisolone was evaluated un the GLUCOVID and Steroids-SARI trial; the use of hydrocortisone by the CAPE-COVID trial and REMAPCAP trial; while dexamethasone was furtherly examined by the DEX-COVID and CoDEX trials. The WHO conducted a comprehensive meta-analysis of these trials, providing a high level of evidence for the efficacy of corticosteroids in patients hospitalized for COVID-19 requiring respiratory support. Particularly, in critically ill patients, systemic corticosteroids compared with no corticosteroid therapy reduced the risk of 28-day mortality (RR 0.80, 95% CI 0.70–0.91) [61]. A recent Italian study, the MEDEAS trial, compared the use of dexamethasone and methylprednisolone in patients hospitalized for COVID-19 pneumonia with respiratory failure. No difference in 30-day mortality was demonstrated between the two groups of treatment; however, it has been found that in more severe patients (P/F < 200) methylprednisolone reduces the ICU admission rate and CRP values more than dexamethasone [62].

*Inhibitors of IL-1 signaling:* IL-1 is one of the major pro-inflammatory cytokines. It comprises two types of ligands, IL-1α and IL-1β, of which IL-1β plays a major role with systemic effects. Higher levels of IL-1β are associated with the severity of COVID-19 infection in critically ill patients [63]. Two inhibitors of the IL-1 pathway have been adopted in rheumatologic diseases: *anakinra*, a recombinant IL-1 receptor antagonist, which is effectively used to treat autoinflammatory diseases, and *canakinumab*, which is a human monoclonal antibody-neutralizing IL-1β [64]. The effects of anakinra are not limited to the treatment of rheumatologic diseases, as it reduces mortality in septic patients experiencing cytokine storm, as demonstrated in randomized clinical trials [65]. Several studies have also investigated the clinical effects of anakinra on COVID-19. An Italian retrospective cohort study showed that high-dose anakinra could be used safely and improved respiratory function in patient with ARDS who were treated with non-invasive mechanical ventilation [66]. Another French prospective cohort study which enrolled patients with severe COVID-19 pneumonia in the ICU demonstrated that anakinra reduced both the need for mechanical ventilation and the mortality without serious side-effects [67]. A prospective cohort study from the Netherlands showed the efficacy of anakinra treatment in reducing clinical signs of hyperinflammation in patients with COVID-19 after 28 days of treatment [68]. Anakinra has, thus, been approved for the treatment of COVID-19 adults with pneumonia requiring supplemental oxygen and who are at risk of developing severe respiratory failure. On the contrary, a limited number of studies have been conducted on the efficacy of canakinumab. Two Italian observational studies suggested that canakinumab treatment may have therapeutic potential in non-ICU patients with mild or severe COVID-19, such as improving oxygenation [69]. Therefore, further validation of the efficacy and safety is needed with controlled trials for this therapeutic strategy.

*Inhibitors of IL-6 signaling:* The pro inflammatory cytokine IL-6 can induce both systemic and local inflammation. In the lungs, IL-6 induces macrophage activation, endothelial leakage and liquid extravasation, while it systemically promotes acute phase protein synthesis and pyrogens activation, which lead to fever. Tocilizumab, an anti-IL-6 receptor, acts by preventing the interaction of IL-6 with its receptors, thereby reducing inflammation and its related symptoms. In COVID-19 infection, an increased serum level of IL-6 has been found to be associated with the severity of disease, including the risk of respiratory failure and death [70]. The large-scale RECOVERY56 and REMAP-CAP studies demonstrated the efficacy of tocilizumab compared with standard care on mortality (Odds ratio 0.86 (CI 95% 0.79–0.95) in patients requiring oxygen therapy [71].

*Tyrosine kinase inhibitors* (TKI) have pleiotropic effects on the immune response given their ability to block many immune effector responses and cytokine pathways. Most TKIs show a considerable safety profile and are adopted in systemic inflammatory diseases, such as connective tissue disease. Janus tyrosine kinase (JAK) is an intracellular tyrosine kinase that mediates signals from cytokines, hormones and growth factors. This enzyme transduces intracellular signals from receptors on the surface for a variety of cytokines and growth factors involved in hematopoiesis, inflammation and immune function. Particularly, JAK phosphorylates and activates the signal transducers and activators of transcription (STAT), which induce gene expression within the cell. The Janus kinase-signal transducer and activator of transcription (JAK/STAT) pathway is commonly involved in various cytokine activation processes. JAK inhibitors, including ruxolitinib and baricitinib, are able to effectively suppress cytokine storm [59]. Baricitinib partially inhibits the enzymatic activity of JAK1 and JAK2, thereby reducing the phosphorylation and activation of STATs. Moreover, JAK inhibitors might also impede the entry and proliferation of SARS-CoV-2. Indeed, AP2-associated protein kinase-1, another target of JAK inhibitors, is a key regulator involved in viral endocytosis and intracellular transport through ACE2 [72]. Several studies have proposed that the JAK/STAT signaling inhibition may be a valuable preventive or therapeutic option for COVID-19. An observational, longitudinal trial showed that baricitinib prevents the progression to the severe form of COVID-19 by modulating the patients’ immune landscape [73]. A multi-centered retrospective study demonstrated that baricitinib reduced the rate of ICU admission and fatality rate in patients, meanwhile increasing the discharge rate [74]. Baricitinib has been authorized by the FDA for its emergency use in treating COVID-19 in patients with severe or critical illness, as it has been demonstrated to reduce mortality, the duration of mechanical ventilation and hospital length of stay.

*IFN-based therapy:* During a viral infection, the interaction between the viral genetic material and the immune system results in the activation of numerous downstream cascade responses, including the production of IFN-stimulating genes and IFN type 1, which can directly inhibit viral replication, as well as activate the cell-mediated response against the virus [63]. For these reasons, the possibility of administering exogenous IFN type 1 has been considered to assist the endogenous response to viral infection and allow more rapid clearance of the virus. Preventing a delayed or inadequate IFN type 1 response to viral infection could be helpful in avoiding the progression of inflammation characterized by cytokine storm and, consequently, reduce potential lung damage. The use of recombinant IFN-α or IFN-β as a treatment in SARS, MERS and now COVID-19 has been a subject of debate. Many researchers attempted to administer type 1 IFNs, such as IFN α, β and κ, in the early phase of COVID-19. Two in vitro studies have already demonstrated that SARS-CoV-2 has greater sensitivity to type 1 IFNs compared with SARS-CoV. In these studies, pre-treatment with IFN-α or IFN-β drastically reduced viral titers [75]. Several clinical trials to evaluate IFN type 1 as a single or combination therapy in COVID-19 have been registered across the world. Particularly, a randomized, double-blind, placebo-controlled, phase 2 pilot study on inhaled IFN-β1a (SNG001) as a single agent demonstrated that patients who received SNG001 had greater odds of clinical improvement and recovered more rapidly from SARS-CoV-2 infection than patients who received the placebo, providing a strong rationale for further trials [76]. Additional results from the ongoing clinical studies, as well as the development of animal models, will offer a more instructive answer on the safety and efficacy of type 1 IFN as a therapy in COVID-19.

IFN-γ, a type 2 interferon, is secreted by several immune cells and plays an important role in stimulating the innate response against viral infection. Its administration has been evaluated in subjects with persistent high viral loads and poor clinical condition, with secondary infectious complications in some case series showing the ability to reduce viral load without leading to signs of hyperinflammation. Intriguingly, emapalumab, a monoclonal antibody targeting IFN-γ, has been tested in COVID-19 patients, starting from the positive results in hemophagocytic lymphohistiocytosis (HLH), a condition with elevated serum levels of IFN-γ, which leads to cytokine storm as it occurs during COVID-19 [77]. Since the contribution of IFN-γ in cytokine storm as mentioned above, emapalumab has been considered as a potential treatment in COVID-19, but no significant data from trials are yet available.

Treatment with IFNs has also been studied in combination with other drugs, such as antivirals or corticosteroids, through intravenous, subcutaneous or inhaled nebulization administration [71].

*TNF-α inhibitors:* TNF-α is a key proinflammatory cytokine contributing to various acute and chronic inflammatory pathologies. Anti-TNF agents, such as etanercept (a soluble TNFα receptor fusion protein), infliximab and adalimumab (monoclonal antibodies targeting TNFα), are commonly used for the treatment of rheumatologic diseases. Thanks to their ability to control cytokine storm, these drugs have also been proposed for the management of COVID-19 in some ongoing clinical trials [78]. Although preliminary results seem to suggest their efficacy as a potential treatment for COVID-19, no sufficient data are available [18].

*Convalescent plasma:* In the absence of specific therapy, especially in the first phase of the pandemic, great interest has been directed towards convalescent plasma collected from donors who have survived COVID-19 and whose plasma contains neutralizing antibodies against SARS-CoV-2. More than 100 clinical trials have been started using this therapeutic approach, and convalescent plasma has been successfully used as either prophylaxis or treatment to provide immediate passive immunity [79]. As previously experienced for other viral infections, providing an immediate humoral response in patients who have not yet developed antibodies can help in reducing the viral load together with stimulating a specific T-cell response.

*Intravenous Immunoglobulins:* Intravenous immunoglobulins (IVIGs) contain the pooled polyclonal immunoglobulin G (IgG) supplied from the plasma of healthy blood donors. IVIGs can elicit passive immunity and exert anti-inflammatory and immunomodulatory effects that can increase patient survival in severe infections. In fact, it has been known that natural anti-cytokine autoantibodies, such as those against IL-1, IL-6 and IFN-γ, can be detected in IVIGs, and that many of these anti-cytokine autoantibodies are neutralizing antibodies, so they may be responsible for the anti-inflammatory effect of IVIGs in inflammatory and autoimmune disorders. Previous anecdotal positive experiences have suggested the use of high doses of IVIG in patients with severe COVID-19 infection. In a randomized, controlled trial including 84 severe COVID-19 patients, IVIG treatment proved able to shorten the length of the ICU and hospital stay [80].

*Monoclonal antibodies targeting the spike protein* are pharmacological molecules specifically designed to target the SARS-CoV-2 spike protein. They have previously shown a high neutralizing activity. Various monoclonal antibodies or combinations thereof have been approved worldwide for the treatment of SARS-CoV-2 infection in patients who do not require oxygen supplementation but who are at a high risk of developing severe forms. Tixagevimab, cilgavimab, regdanvimab, casirivimab, imdevimab and sotrovimab are all monoclonal antibodies directed towards the spike protein of SARS-CoV-2 to prevent viral entry in the host cells [81]. The emergence of SARS-CoV-2 variants, particularly the Omicron variants, causes mutations in the epitopes of the spike proteins. Unfortunately, this has consequently reduced the efficacy of most of the monoclonal antibodies, which are directed against the spike protein of the original SARS-CoV-2. Bebtelovimab is a recently developed monoclonal antibody that also has neutralizing activity against Omicron variants [82].

*Other therapies* have been adopted in COVID-19 patients, although only in small groups or selected populations. Among these cell-based therapies are PD-1 Checkpoint inhibitors and hydroxychloroquine.

*Cell-based therapy:* The use of mesenchymal stem cells (MSCs) in the treatment of severe forms of COVID-19 has been tested. MSCs appear to have immunoregulatory and antiapoptotic activities, as well as the ability to induce angiogenesis and tissue healing. It appears that MCSs are able to both stimulate a broad response against the virus and produce anti-inflammatory mediators in response to IFN. In addition, the production of angiopoietin 1 and keratinocyte growth factor by MSCs has shown effects in the repair of the alveolar-capillary membrane. The activity of MSCs might be particularly effective because, after venous injection, they show homing in the lungs and tend to migrate to the most damaged tissues, where they stimulate lung repair and damaged tissue regeneration [83].

*PD-1 Checkpoint Inhibitors:* Lymphocytes exhaustion is one of the characteristics of COVID-19. PD-1 Checkpoint inhibitors might help in reversing the anergy of lymphocytes. Signaling pathways regulated by PD-1/PD-L1 bounding also play a role in balancing protective immunity, and they can be involved in the activation of cytokine storm that leads to severe sepsis. Nivolumab (anti PD-1) and BMS-936559 (anti PD-L1) have completed phase-Ib randomized studies for severe sepsis, so they might be useful in treatment of severe COVID-19 [59].

*Hydroxychloroquine* is an antimalarial agent commonly used in rheumatology practice based upon its immunomodulatory effects. At the beginning of the COVID-19 pandemic, hydroxychloroquine was commonly adopted in the treatment of the disease. However, subsequent systematic reviews and meta-analysis have concluded that hydroxychloroquine has no clinical effect on patients with COVID-19 and it is now not recommended [84].

### 3.6. Vaccines

**Figure 3:**
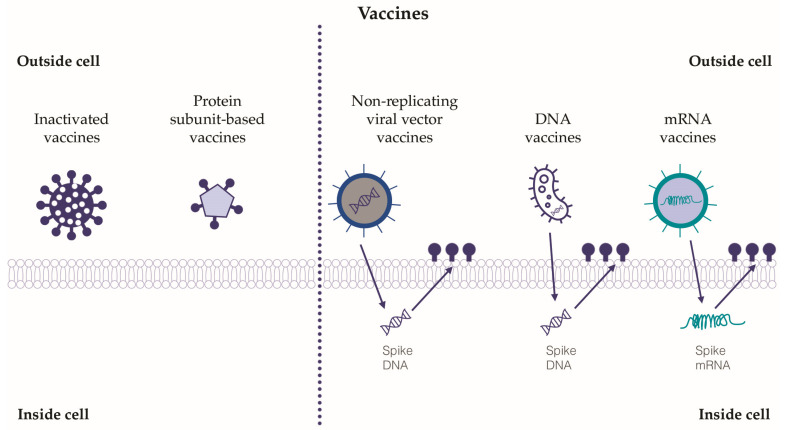
The relevant classes of tested vaccines against SARS-CoV-2. Five classes of vaccines against SARS-CoV-2 have been tested, and some have been approved for use in humans. Inactivated vaccines and protein subunit-based vaccines exert their action outside the target cells, whereas non-replicating viral vector vaccines, DNA and mRNA vaccines need to act inside the target cells in order to adopt the cellular transcription and translation systems. The immune response and immunization induced by viral vector vaccines, DNA and mRNA vaccines seem to be more relevant than those induced by inactivated and protein subunit-based vaccines.

The development of vaccines against SARS-CoV-2 has represented a global challenge in the field of pharmacological research. Hundreds of vaccines have been designed, but only a few of them have passed the necessary approval stages for administration in humans. For the purposes of this review, the relevant classes of vaccines tested will be described, while a detailed analysis or comparison of the efficacy and safety of the vaccines currently on the market is beyond the current paper’s scope (Figure 3).

It has been demonstrated that the imbalance between increased CD3+ CD8+ T cells and decreased CD14+ HLA-DR+ monocytes may worsen COVID-19 infection; thus, one of the goals of vaccines against SARS-CoV-2 has been to enhance an effective immune response, both cellular and humoral, without creating such an imbalance [85,86].

*Inactivated vaccines*: Several inactivated SARS-CoV-2 vaccines have been developed [85]. Inactivated vaccines are obtained from coronavirus after its inactivation due to exposure to physical or chemical inactivating agents, such as UV rays, formalin and formaldehyde. To be effective and able to induce a consistent immune response, they have to be combined with adjuvants [87,88]. Inactivated vaccines have proven to be safe, well tolerated and with a discrete efficacy in the induction of the humoral response, although the immunization period might be shorter than with other types of vaccine [89].

*Non-replicating viral vector vaccines*: Inactivated adenoviruses are commonly adopted as vectors in non-replicating viral vector vaccines [90]. This approach has been tested for the development of vaccines against SARS-CoV-2. Non-replicating viral vector vaccines can induce a strong humoral and cellular immune response, and they show good safety and immunogenicity, guaranteeing high titers of antibodies directed against the spike protein of SARS-CoV-2 [91]. However, adenoviruses are among the most common agents of respiratory infections; thus, a limitation of adopting this virus as a vaccine vector might lie in previous immunization to strands of adenovirus similar to the one adopted as a vector with a consequent decrease in efficacy [92]. For this reason, commercially available non-replicating viral vector vaccines for SARS-CoV-2 are based on non-human specific adenovirus or less-common human-specific adenoviral vectors, i.e., Ad26 and Ad35 [93,94].

*DNA vaccines*: DNA vaccines act through insertion into the host cells of genes encoding a specific antigen. More in detail, DNA plasmids are used as vectors to insert the genes, especially in antigen-presenting cells (APC). Electroporation, temporarily creating pores in the cellular membranes, allows the injection of plasmid DNA into the target cells. Once injected, the genetic material can reach the cell nucleus and subsequently be transcribed and directly expressed by the host cell with great production of the target protein [75,77]. DNA vaccines are non-infectious, thermally stable, and can guarantee good immunization. Clinical trials have been performed for a DNA vaccine containing DNA encoding, both for the whole S-protein or a fraction of it (S-protein without S2), and the results show their efficacy in the production of high titers of neutralizing antibodies [95].

*mRNA vaccines*: Specific mRNA molecules encoding protein antigens can be delivered inside lipid nanoparticles, which at the same time, act as mRNA carriers and adjuvants [96]. In mRNA vaccines, once the specific mRNA molecule enters the target host cell, it is translated in the active protein directly inside the cytoplasm, without entering the nucleus. These vaccines have proved able to activate both the cellular and humoral response, without any risk of insertional mutations (since there are no interactions with the nucleus) or infection (since there are no viral vectors) [97]. Long-term immunity can be obtained after immunization with mRNA vaccines; more in detail, data from clinical trials have suggested one or more boosters after the first immunization in order to maintain a protective immune response for a long time, ensuring high antibody titration [98]. Although first tested to produce a vaccine against respiratory syncytial virus, HIV, influenza virus and Ebola virus [99,100], the currently available mRNA vaccines against SARS-CoV-2 are the most diffuse to fight the pandemic around the world. Intriguingly, the mRNA-1273-based vaccine (developed by Moderna^©^) encodes the S2 protein antigen, which is composed of SARS-CoV-2 glycoprotein with the transmembrane anchor and S1-S2 cleavage site [100]. The BNT162b1 and BNT162b2 vaccines (developed by Pfizer^©^ and BioNTech^©^) encode for the secreted receptor-binding-domain (BNT162b1) of SARS-CoV-2 and the membrane-anchored full-length S-protein (BNT162b2) [101]. Notably, mRNA vaccines can induce a higher antibody titer than the one obtained after recovering from COVID-19 [102]. Moreover, mRNA vaccines seem to be more easily re-projectable towards emerging viral mutations [103].

*Protein subunit-based vaccines*: Genes encoding major viral antigenic components are cloned to be expressed in selected cells (bacterial or mammalian), and then purified. After purification, the recombinant proteins can be used as vaccines. The most known of these vaccines against SARS-CoV-2 is NVX-CoV2373 (produced by Novavax^©^), which contains a recombinant engineered full-length spike glycoprotein with a high binding affinity for ACE2 receptors [104]. Protein subunit-based vaccines are safe and cost-effective, although they need a large number of adjuvants in order to enhance antigen presenting cells to uptake the recombinant protein [105].

## 4. Conclusions and Future Perspectives

The immune system plays a key role in hampering the evolution of COVID-19 towards severe forms of the disease. Both innate and adaptive immune responses are activated during SARS-CoV-2 infection. An out-of-control immune response and hyperinflammatory state resulting from cytokine storm leads to deleterious effects that are the basis of severe complications, such as ARDS, multi-organ failure and death. The development of therapies based on the modulation of the immune response that attempt to block or curb cytokine storm appear to be the most effective available weapons against COVID-19. Finally, the development and use of effective vaccines to protect against the most severe forms of the disease and reduce mortality is now recognized as the best viable strategy for the fight against the pandemic.

## Figures and Tables

**Table 1:** Therapeutic agents that have been tested for the treatment of COVID-19. For every agent, the therapeutic class, molecule, target and mechanism of action have been described. A quality of evidence for the efficacy has also been specified, according to data available from trials and observational studies already available in the literature. IL, interleukin; TNF, tumor necrosis factor; JAK, Janus kinase; STAT, signal transducers and activators of transcription; IFN, interferon; NF-kB, nuclear factor kappa; ACE2, angiotensin-converting enzyme 2.

Therapeutic Strategies Experimented in COVID-19				
Therapeutic Class	Molecule	Target	Evidence of Efficacy from Trials	Mechanism of Action
Corticosteroids	Dexamethasone, Methylprednisolone, Hydrocortisone	T cells; Macrophages	High	* Synthesis inhibition of Th1 and macrophage pro-inflammatory cytokines (IL-1β, IL-2, IL-6, TNF-α, and IL-17) * Gene transcription and protein synthesis of NF-kB and lipocortin-1 inhibitors
Inhibitors of IL-1 signaling	Anakinra	IL-1 receptor	Moderate	* Reduction of the cascade release of proinflammatory cytokines (TNF, IL-6, IL-8); * Reduction of fever by modulation of thermoregulatory center in the brain
	Canakinumab	IL-1β	Mild	* Reduction of the cascade release of proinflammatory cytokines (TNF, IL-6, IL-8); * Reduction of fever by modulation of thermoregulatory center in the brain
Inhibitors of IL-6 signaling	Tocilizumab	IL-6 receptor	Moderate/High	* Reduction of macrophage activation; * Reduction of endothelial leakage and liquid extravasation; * Reduction of acute phase protein synthesis and pyrogens activation.
Tyrosine kinase inhibitors	Baricitinib	JAK 1 and JAK 2	Moderate/High	* Inhibition of the enzymatic activity of JAK1 and JAK2; * Reduction of the phosphorylation and activation of STATs; * Interference with viral endocytosis and intracellular transport through ACE2; * Suppression of cytokine storm
IFN-based therapy	Recombinant IFN α and β	Type 1 IFN receptor	Mild/Moderate	* Inhibition of viral replication; * Activation of the cell-mediated response against the virus
	Emapalumab	IFN-γ	Low	* Stimulation of the innate response against viral infection
TNF-α inhibitors	Etanercept	TNFα receptor	Very Low	* Suppression of cytokine storm
	Infliximab and Adalimumab	TNFα	Very Low	* Suppression of cytokine storm
Convalescent plasma	Neutralizing antibodies	SARS-CoV-2	Moderate/Low	* Passive humoral response
Intravenous Immunoglobulins	Anti-cytokine autoantibodies (i.e. against IL-1, IL-6, and IFN-γ)	IL-1, IL-6, IFN-γ	Moderate/Low	* Anti-inflammatory and immunomodulatory effects
Monoclonal antibodies targeting the spike protein	Tixagevimab, casirivimab, sotrovimab	Spike protein of SARS-CoV-2	Moderate	* Prevention of viral entry in the host cells

## Data Availability

Not applicable.
